# Statin Treatment-Induced Development of Type 2 Diabetes: From Clinical Evidence to Mechanistic Insights

**DOI:** 10.3390/ijms21134725

**Published:** 2020-07-02

**Authors:** Unai Galicia-Garcia, Shifa Jebari, Asier Larrea-Sebal, Kepa B. Uribe, Haziq Siddiqi, Helena Ostolaza, Asier Benito-Vicente, César Martín

**Affiliations:** 1 Fundación Biofisika Bizkaia, Barrio Sarriena s/n, 48940 Leioa, Spain; ugalicia001@ehu.eus (U.G.-G.); Alarrea001@ehu.eus (A.L.-S.); ofbmaplc@ehu.es (H.O.); asier.benito@ehu.eus (A.B.-V.); 2 Biofisika Institute (UPV/EHU, CSIC), Barrio Sarriena s/n, 48940 Leioa, Spain; sjebari001@ikasle.ehu.eus; 3 Department of Biochemistry and Molecular Biology, Universidad del País Vasco UPV/EHU, Apdo. 644, 48080 Bilbao, Spain; 4 Center for Cooperative Research in Biomaterials (CIC biomaGUNE), Basque Research and Technology Alliance (BRTA), Paseo de Miramon 182, 20014 Donostia San Sebastián, Spain; kbelloso@cicbiomagune.es; 5 Harvard Medical School, Harvard University, 25 Shattuck St, Boston, MA 02115, USA; Haziq_Siddiqi@hms.harvard.edu

**Keywords:** statin, type 2 diabetes mellitus, clinical trial, insulin resistance, microRNA

## Abstract

Statins are the gold-standard treatment for the prevention of primary and secondary cardiovascular disease, which is the leading cause of mortality worldwide. Despite the safety and relative tolerability of statins, observational studies, clinical trials and meta-analyses indicate an increased risk of developing new-onset type 2 diabetes mellitus (T2DM) after long-term statin treatment. It has been shown that statins can impair insulin sensitivity and secretion by pancreatic β-cells and increase insulin resistance in peripheral tissues. The mechanisms involved in these processes include, among others, impaired Ca^2+^ signaling in pancreatic β-cells, down-regulation of GLUT-4 in adipocytes and compromised insulin signaling. In addition, it has also been described that statins’ impact on epigenetics may also contribute to statin-induced T2DM via differential expression of microRNAs. This review focuses on the evidence and mechanisms by which statin therapy is associated with the development of T2DM. This review describes the multifactorial combination of effects that most likely contributes to the diabetogenic effects of statins. Clinically, these findings should encourage clinicians to consider diabetes monitoring in patients receiving statin therapy in order to ensure early diagnosis and appropriate management.

## 1. Introduction

Statins are a guideline-directed, first line therapy for prevention of primary and secondary cardiovascular disease (CVD), which is the leading cause of mortality worldwide [1,2]. Although the principal mechanism of the action of statins is inhibition of 3-hydroxy-3-methyl-glutaryl coenzyme-A (HMG-CoA) reductase, statins have been implicated in several other beneficial pleiotropic effects including improving endothelial function, stabilization of atherosclerotic plaques and anti-inflammatory activities [3]. Despite the safety and relative tolerability of statins, observational studies [4,5,6,7,8], clinical trials [9,10] and meta-analyses [11,12,13,14,15,16] have found that statins can increase the risk of new-onset type 2 diabetes mellitus (T2DM). These studies implicated statins in negatively impacting insulin sensitivity, decreasing secretion by pancreatic β-cells and increasing insulin resistance [11,17,18]. While the lipid-lowering mechanism of statins is relatively well understood, the mechanisms underlying statin-induced T2DM development seem to be multifactorial and remain unclear. Among experimental studies, multiple works have indicated that statins diminish pancreatic β-cell function via Ca^2+^ signaling pathways impairment [19,20], compromise insulin signaling and down-regulate the insulin-responsive glucose transporter 4 (GLUT-4) [21,22]. In addition, it has also been described that statins impact on epigenetics may also contribute to statin-induced T2DM via differential expression of microRNAs [23].

This review focuses on the evidence and mechanisms by which statin therapy is associated with the development of T2DM. Here, we will describe the existing data from clinical studies as well as experimental results that shed some light on the mechanisms of this association.

## 2. Primary Action of Statins: Cholesterol Biosynthetic Pathway

**Figure 1:**
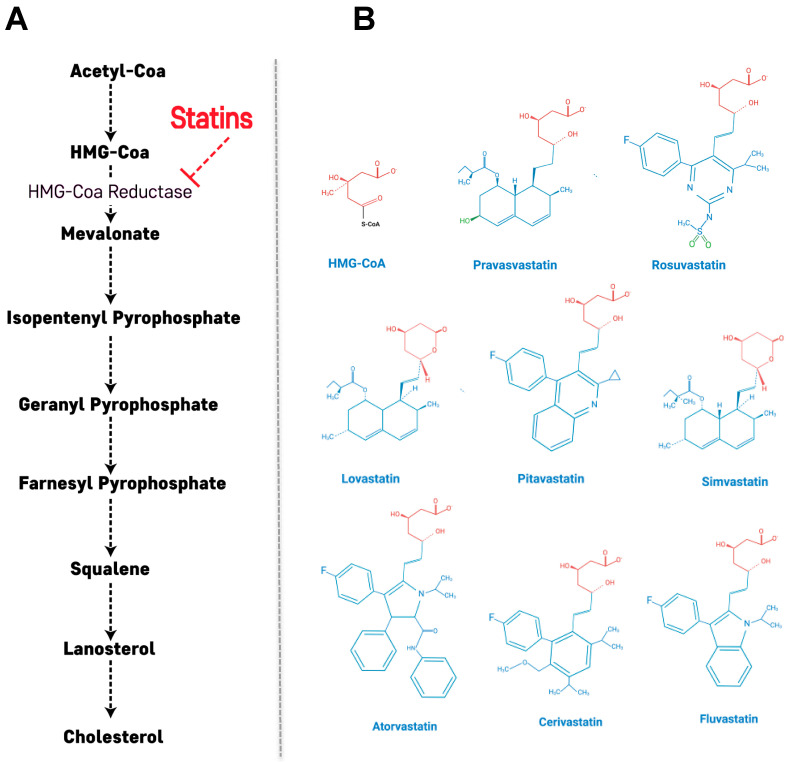
Statin-induced inhibition of the mevalonate pathway and structure of statins. (**A**) Inhibition of HMG-CoA reductase significantly blocks the production of mevalonate, a necessary precursor for cholesterol synthesis. Mevalonate is the building block for a variety of other compounds. (**B**) Structural formulas of statins and HMG-CoA. The HMG-like moiety (in red) is conserved in all statins. The polar substituents responsible of pravastatin and rosuvastatin are colored in green.

Statins are reversible and competitive inhibitors of HMG-CoA reductase, which is the rate-determining enzyme in the cholesterol biosynthetic pathway [24]. The HMG-like portion of statins, which is a modified 3,5-dihydroxyglutaric acid moiety, is structurally similar to HMG-CoA and causes the inhibition of HMG-CoA reduction reactions [25]. Through this mechanism, the mevalonate pathway is inhibited along with a consequent decrease in downstream products and cholesterol synthesis (Figure 1A). In addition, this statin-mediated decrease in intracellular cholesterol content leads to up-regulation of the LDL receptor (LDLR) in the liver and peripheral tissues, resulting in decreased blood LDL cholesterol (LDL-C) [26]. LDLR is the primary route by which LDL-C is removed from circulation, and its synthesis has been shown to be inversely correlated to the amount of cholesterol synthesized by a cell [27]. Through the action of statins, the cellular cholesterol concentration decreases, stimulating production of more LDLR and promoting LDL-C removal from the bloodstream, ultimately reducing CVD risk [27].

Statins are classified according to their hydrophobicity into hydrophilic statins (pravastatin and rosuvastatin) and lipophilic statins (atorvastatin, cerivastatin, fluvastatin, lovastatin, pitavastatin and simvastatin) [28,29]. The solubility and pharmacological properties of statins are determined by the substituents on the ring attached to the active moiety [29]. Hydrophilicity originates from polar substituents added to the active site while the addition of nonpolar substituents leads to lipophilicity [25,29] (Figure 1B). Although the target of both types of statins is HMG-CoA reductase, the inhibitory mechanisms are distinct. Hydrophilic statins target the liver more efficiently because their uptake is carrier-mediated, while lipophilic statins passively diffuse through the hepatocellular membrane and similarly are also able to diffuse in extrahepatic tissues, thus showing reduced hepatoselectivity [29,30]. Their diffuse influence on extrahepatic tissues may explain the higher incidence of adverse effects observed with lipophilic statins. The notable exception to this is rosuvastatin, which is a hydrophilic statin but has a similar activity profile to lipophilic statins [31].

## 3. Beneficial Effects of Statins on Diabetic Complication and/or Inflammation in T2DM

There are many factors that contribute to the development of atherosclerotic cardiovascular disease, the main mortality cause in T2DM patients. These include dyslipidemia, increased oxidative stress, enhanced protein glycation or chronic inflammatory state all of them worsen in T2DM [32]. Statins are the gold standard treatment for the prevention and management of cardiovascular disease and their use in T2DM patients is recommended by The American Diabetes Association 2019 guidelines [33]. In addition to the reduction of cholesterol levels and dyslipidemia improvement by reducing lipoprotein levels in plasma, the pleiotropic effects of statins reduce high sensitive C-reactive protein and other pro-inflammatory markers [34], improve endothelial function and reduce oxidative stress [35], which together contribute to a significant CVD reduction in T2DM patients.

Several clinical trials have pointed out the beneficial effects of statins in diabetic patients [36]. The collaborative atorvastatin diabetes study (CARDS) showed nearly 40% reduction in relative risk of cardiovascular events in diabetic patients aged 45–70 years old with high cholesterol levels and treated with atorvastatin during 4 years [37]. A meta-analysis of 14 randomized trials including more than 18,000 patients confirmed the beneficial effects of statins in diabetic patients showing a 21% reduction in major vascular events per mmol/L LDL-C reduction [38]. Further studies, confirmed the benefits of statin treatment in diabetic patients independently of LDL-C baseline [39].

Unfortunately, in some cases, statin treatment leads to adverse effects such as the decreased insulin sensitivity shown by atorvastatin, simvastatin and rosuvastatin [40]. For atorvastatin and simvastatin, one proposed explanation is that the higher diffusion rate of lipophilic statins to the intracellular space can interfere with cellular processes, leading to decreased intracellular insulin secretion in response to glucose [41]. For rosuvastatin, despite its hydrophilicity, the higher affinity and efficient transport of rosuvastatin into cells, which can underlie it effects on insulin sensitivity [29].

## 4. Statin Therapy and Risk of Developing T2DM: Observational Studies, Clinical Trials and Meta-Analysis

Statins, discovered in the early 70s and commercially available in the mid-80s, have well-characterized benefits in terms of lowering LDL-C and cardiovascular risk reduction. However, 20 years after becoming commonly prescribed, findings from observational studies showed an increased T2DM risk upon statin administration in several populations. Despite the considerable variability among these studies and the statin administered, hazard ratios (HR) were statistically significant ranging from 1.19 to 1.57, after follow-up durations of 3–6 years [4,6,7]. Observational studies carried out in Canada, Taiwan and Ireland examining the association between statin administration and T2DM development, showed 10–22%, 15% and 20% increases in the risk of T2DM associated with statin therapy, respectively [42,43,44]. Later on, the effects of statin treatment on the risk of T2DM and hyperglycemia deterioration were assessed in the metabolic syndrome in men (METSIM) study cohort, which found that statin therapy was associated with a 46% increased risk of T2DM along with worsening of hyperglycemia [45]. In addition, the study found statin use to be associated with a 24% reduction in insulin sensitivity and a 12% decrease in β-cell count compared to individuals not taking statin therapy [45]. Notably, treatment with both simvastatin and atorvastatin was associated with reductions in insulin sensitivity and secretion in a dose-dependent manner [5].

Collectively, statin randomized control trials (RCT) were designed and, large, long-term, double blind, placebo-controlled studies were conducted to evaluate the effects of statins in a variety of clinical situations. Although most statin RCTs, including the largest statin RCT trial, were designed primarily to evaluate efficacy in a variety of clinical situations, several RCTs also evaluated the relationship between stain treatment and T2DM development. Among them, the justification for the use of statin in prevention: an intervention trial evaluating rosuvastatin (JUPITER), study of the effectiveness of additional reductions in cholesterol and homocysteine (SEARCH) and Cholesterol Treatment Trialists trials were un-confounded regarding the intervention and aimed to recruit at least 1000 participants with treatment duration of at least 2 years.

The justification for the use of statin in prevention: an intervention trial evaluating rosuvastatin (JUPITER) trial showed a small but significant increase in diabetes incidence rates in patients who received statin treatment when compared to placebo over a median of 1.9 years (absolute increase of 0.6%; relative increase of 24%; *p* = 0.01) [46]. Subsequent meta-analyses of the available randomized controlled trials showed that standard statin dose regimens were associated with a proportional increase of about 10% in reported T2DM. According to the results of the JUPITER trial, treatment with high statin concentrations resulted in a further increase by 10% [16,47]. In addition, a post-hoc analysis of the JUPITER trial showed that participants with one or more major diabetes risk factor were at higher risk of developing T2DM than were those without a major risk factor. Of note, however, benefits of statin therapy exceeded the diabetes hazard even in participants at high risk of developing diabetes [10]. In patients who had risk factors for diabetes (e.g., elevated body-mass index or HbA1c, or impaired fasting glucose), the excess of T2DM diagnoses appeared soon after the start of statin therapy, and did not appear to get larger as treatment continued [10,48,49].

Another RCT carried out by the study of the effectiveness of additional reductions in cholesterol and homocysteine (SEARCH) collaborative group found that the simvastatin treatment was associated with a dose-dependent increased risk of diabetes, with diabetes found in 11.6% participants who received 80 mg simvastatin compared to 10.9% in participants receiving 20 mg simvastatin [50]. Collectively, the findings of multiple RCTs indicate that statin therapy may lead to the development of diabetes [51]. Although results from individual RCTs have shown substantial variability in the association between statin therapy and incident diabetes, they generated a large amount of data that could be more powerfully analyzed in meta-analysis. For the most relevant insights, meta-analyses that compile data from several RCTs represent a powerful tool for understanding the impacts of statin therapy.

Consistent with the aforementioned RCTs, The Cholesterol Treatment Trialists’ Collaborators meta-analysis (CTT) showed that LDL-C reduction is associated with a 21% reduction in the incidence of any major vascular event in both patients with or without diabetes [38]. In the study, randomized trials were eligible for inclusion if: (i) the main effect of at least one of the trial interventions was to modify lipid levels; (ii) the trial was unconfounded with respect to this intervention (i.e., no other differences in modification of risk factors between the relevant treatment groups were intended) and (iii) the trial aimed to recruit 1000 or more participants with treatment duration of at least 2 years [38]. The study assessed possible variation in the proportional effects of allocation to a statin in different circumstances only for major vascular events. Trial participants were considered to have diabetes if they had a recorded history of diabetes at randomization, and subdivision of diabetes subtypes was done according to the definitions used in the individual trials [38]. The study showed that statins are directly correlated with an increased risk of developing T2DM. Interestingly, multiple meta-analyses have found that the risk of statin-associated T2DM is higher in participants taking higher doses when compared to patients taking lower doses [15,16,47]. Accordingly, the data obtained indicated an excess risk ranging from 9% to 13%, with the highest risk of T2DM seen in patients taking high-intensity statin therapy [13,15,16,43,47,52]. Specifically, a recent meta-analysis showed that atorvastatin 80 mg is associated with the highest risk of T2DM, followed by rosuvastatin and simvastatin 80 mg, indicating that statins have varying effects on the risk of T2DM [53]. Overall, meta-analysis studies found a clear association between diabetes and statins across multiple statins, indicating that the diabetogenic property of statins is a class effect. Most importantly, despite the increase of T2DM, it is important to emphasize that the benefits of statin administration in reducing myocardial infarction, stroke and cardiovascular deaths in high CVD risk patients are enough to warrant statin treatment, although T2DM prevention and screening is important to take into consideration.

As listed above, clinical trials, meta-analyses and observational studies highlight that patients who received statin treatment had a 10–12% increase in T2DM risk [17]. However, the risk is even higher in patients receiving high-intensity statin therapy and among patients with pre-existing risk factors for diabetes. Recent studies indicate a clear correlation between statin type and treatment intensity with T2DM development. Specifically, pravastatin 40 mg/day treatment has been associated the lowest risk of T2DM, while rosuvastatin 20 mg/day and atorvastatin 80 mg/day treatment are associated with increased risks of T2DM. Between rosuvastatin and atorvastatin, rosuvastatin has been associated with the higher risk of T2DM [14].

However, even if statin type and treatment intensity clearly correlate with T2DM development, individual’s risk factors should not be overlooked. Development of T2DM during statin treatment is more frequent among individuals with pre-existing risk factors, including increased adiposity, predisposing dietary patterns, sedentary lifestyle, psychosocial factors and previous medical history [54], as well as age and gender [55]. In fact, for patients with none to 1 risk factor, the incidence of T2DM is similar between those receiving high dose and moderate dose of statins (3.22% and 3.35%, respectively). Conversely, for patients with 2–4 risk factors the incidence is 14.3% in the high dose group and 11.9% in the moderate dose group [17].

## 5. Proposed Mechanisms for T2DM Development Induced by Statins

**Figure 2:**
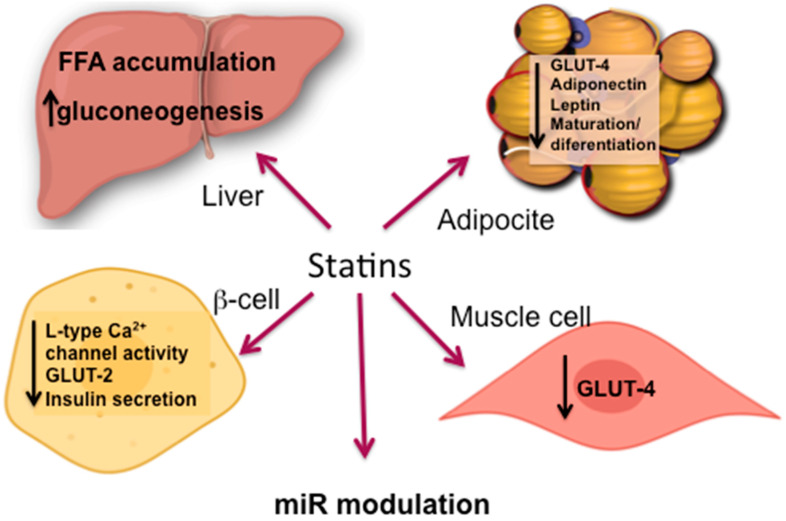
Principal mechanisms for T2DM development induced by statins.

Overall, the mechanisms by which statin treatment induces T2DM are not fully understood, but both on-target and off-target effects may be involved. Among these, inhibition of the mevalonate pathway results in a reduction in several cellular biosynthetic pathways including those involved in glucose homeostasis [56]. Over time, chronic statin treatment increases gluconeogenesis by upregulating gene expression of key enzymes that increase glucose production in the liver [57]. In addition, it has been shown that statins can impair the insulin signaling pathway as well as downregulate the GLUT-4 transporter, which is responsible for the uptake of glucose in peripheral cells [22,58,59]. Statins can also induce changes in circulating free fatty acids (FFA), changes in hormones such as adiponectin and leptin, impairment of β-cell function, β-cell cell damage and adipocyte maturation/differentiation [17,56,60]. Additional mechanisms involving epigenetic regulation mediated by specific microRNAs have also being involved in the reduction of insulin secretion [56]. These complex pathophysiologic molecular mechanisms of statin-induced T2DM, summarized in Figure 2, are described in more detail in the following sections.

### 5.1. Dysfunctional Effects Caused by Statins in Pancreatic β-Cell

**Figure 3:**
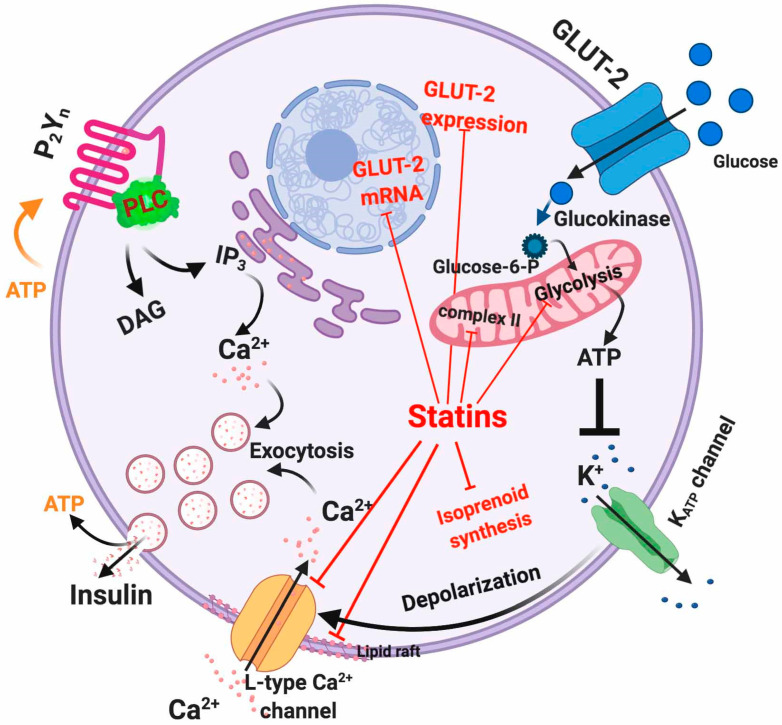
Intracellular actions of statins in β-cells. Red lines indicate the mechanisms affected by statins.

Insulin secretion from pancreatic β-cells is initiated by glucose-induced Ca^2+^ entry controlled by voltage-gated Ca^2+^ channels (Figure 3) [61]. Therefore, maintenance of intracellular Ca^2+^ homeostasis is tightly regulated in order to ensure proper insulin secretion and maintain the integrity of the β-cell physiology [62]. Briefly, glucose uptake activates glycolysis in β-cell thus elevating the [ATP]/[ADP]_i_ ratio [63]. This acts as a signal that closes K_ATP_ channels and depolarizes the plasma membrane, with subsequent activation of voltage-dependent Ca^2+^ channels, entry of extracellular Ca^2+^ and finally insulin exocytosis (Figure 3) [64]. ATP sensitivity of the K_ATP_ channels is modulated by several effectors including PIP_2_ and acyl CoAs [65,66]. Conversely, a decrease in the metabolic signal causes reopening of K_ATP_ channels and suppresses the electrical trigger for insulin secretion, thereby providing feedback regulation of insulin secretion [67]. In addition, ATP and ADP can act as autocrine activators of β-cell purinergic receptors because they are also within insulin exocytosis granules [68]. Indeed, inhibition of both P2X and P2Y purinergic receptors causes a reduction in glucose-induced insulin secretion [69,70,71,72] (Figure 3).

To date, the relationship between statin-mediated inhibition of cholesterol synthesis and impaired L-type Ca^2+^ channel activity remains unclear. However, in vitro studies have indicated that simvastatin can directly inhibit L-type Ca^2+^ channels in rat pancreatic islet β-cells [41]. Specifically, because simvastatin was found to immediately inhibit channel activity, it has been suggested that there is a direct interaction between simvastatin and the channel. In contrast, pravastatin lacks L-type Ca^2+^ channels inhibition, possibly because of its lipophilicity [41]. Alternatively, other authors have suggested that the long-term cholesterol reduction caused by statins can lead to incorrect sorting of membrane lipid-raft bound proteins or conformational changes of the Ca^2+^ channel subunits [73]. More recently, it has been suggested that statins can reduce the membrane potential by inhibiting mitochondrial complex II activity, which causes oxidative stress [74]. These off-target effects of statins have been very recently corroborated by Curry et al. [75] in experiments showing that simvastatin impairs β-cell function by at least two mechanisms: (1) via direct inhibition of K_ATP_ channels in a mitochondria-independent manner and (2) via interference with mitochondrial respiration, thus decreasing cytosolic ATP levels and inhibiting metabolic upregulation of L-type Ca^2+^ channels [75].

As described before, insulin is secreted by β-cells in response to glucose uptake through GLUT receptors (primarily GLUT-1 to 4), with GLUT-2 being the predominant isoform in β-cells [76,77]. GLUT-2 represents a high-affinity and low-capacity glucose transporter [78]. It has been shown that treatment of β-cells with atorvastatin and pravastatin inhibited GLUT-2 expression in a concentration-dependent manner [58]. However, rosuvastatin and pitavastatin showed a slight increase in GLUT-2 expression [58]. In addition to this, it has also been observed in mouse pancreatic β-cell line MIN6 cells that simvastatin treatment diminishes GLUT-2 mRNA and protein expression via a dose-dependent reduction of ATP production [79]. Another mechanism through which statins may interfere with glucose metabolism is the statin-mediated LDLR upregulation that increases cholesterol uptake in the β-cell leading to reduced mRNA and protein expression of GLUT-2, consequently limiting glucose uptake [19,80].

The direct inhibition of the mevalonate pathway by statins reduces the intracellular concentration of isoprenoids, the final products of the pathway. Isoprenoids are essential for G protein posttranslational modification, which is important for insulin granule exocytosis [17]. Interestingly, it has been shown that the glucose-induced insulin secretion by lovastatin in normal rat islets is reduced by co-incubation with mevalonate [81]. The adverse effects of statins are summarized in Figure 3.

### 5.2. Statin Induced IR

The binding of insulin to the insulin receptor (INSR) triggers insulin signaling with the physiologic objective of normalizing high blood glucose levels [82]. Insulin binding induces structural rearrangements in the INSR leading to auto-phosphorylation of tyrosine residues. The downstream events that follow INSR activation include recruitment of several adaptor proteins, facilitating a suitable binding site for insulin receptor substrates (IRSs) [82] that once phosphorylated, trigger several downstream signals [83]. Among them, IRS-1 is phosphorylated and activates different kinases such as Akt, PKC, SIK2, S6K1, mTOR, ERK1/2 and ROCK1 [83,84]. IRS-1 activates PI3K, which in turn, catalyzes the conversion of PIP_2_ to PIP_3_, which activates Akt, among other targets [85]. Akt activation leads to glucose uptake by facilitating GLUT-4 translocation to the plasma membrane [86]. GLUT-4 is an insulin-dependent glucose transporter primarily expressed in adipose tissue, cardiomyocytes and skeletal muscle cells [87].

Akt also promotes glycogen synthesis by inhibiting glycogen synthase [88]. In addition, insulin also triggers several IRS-independent signaling pathways, among them those mediated by heterotrimeric G protein and SOS-growth factor [89].

Several disturbances in insulin signal transduction mediated by statin treatment have been described in different organs and tissues leading to a pathologic insulin resistance. This condition is characterized by a pathophysiologic failure to proper respond to normal circulatory levels of insulin in insulin-sensitive cells, such as adipocytes, skeletal muscle cells and hepatocytes [90]. Below, we review some proposed mechanisms through which statins interfere with the insulin response in each of these tissues.

#### 5.2.1. Adipose Tissue

Recently, evidence that statin treatment impairs the insulin signal transduction process in adipocytes, including INSR, GLUT-4, Akt, some small GTP-binding proteins (G-proteins) and caveolae integrity has been demonstrated. Multiple studies have shown that atorvastatin and lovastatin reduce GLUT-4 expression at the plasma membrane in 3T3L1 adipocytes [91,92] and a similar effect has been described with atorvastatin in mouse-white adipose tissue, thus impairing glucose tolerance [22]. The statin-induced decrease in GLUT-4 translocation to the plasma membrane has been attributed to inhibition of isoprenoid synthesis [22]. In fact, isoprenylation is essential for the correct functioning of several proteins involved in the GLUT-4 translocation process. As previously described, isoprenylation is impaired due to statin-induced inhibition of the mevalonate pathway. In one illustrative example, it has been described that atorvastatin disrupts plasma membrane colocalization of Rab-4 and RhoA through inhibition of geranylgeranyl pyrophosphate synthesis. Rab-4 and RhoA are isoprenoid-dependent proteins, which are involved in the insulin-induced translocation of GLUT-4, thus their atorvastatin-mediated dysfunction may disturb overall insulin signaling [93]. RhoA modulates the activities of IRS-1 in 3T3-L1 adipocytes, in which atorvastatin has been shown to reduce the active membrane fraction of both RhoA and Rab4 [93].

Statins also disrupt the formation of caveolae, plasma membrane microdomains at which GLUT-4 anchors after insulin-stimulated translocation [94]. Furthermore, it has been shown that INSR is highly enriched in adipocyte caveolae [95,96] through interaction of its beta subunit with Caveolin 1 (Cav-1), one of the essential constituents of caveolae. Thus, it has been suggested that Cav-1 stabilizes the insulin receptor at the protein level, acting as a molecular chaperone necessary for proper insulin signaling in adipocytes in vivo [97].

The cholesterol dependence of caveolae in order to acquire their characteristic shape is well known [98]. Caveolae dynamics are tightly regulated by caveolin and cavin proteins. Importantly, cholesterol depletion can disrupt this regulation. The adverse effects of statins within caveolae seem to be partially mediated by the stoichiometric binding of Cav-1 to cholesterol [99] and by cavins, which show an essential cholesterol-dependence for defining caveolar structure [98]. Statin-induced cholesterol depletion leads to proteasomal degradation of cavin-2 and relocation of cavin-1 to the cytosol leading to caveolae disruption [98]. Moreover, statin disruption of caveolar formation seems to reduce secretion of high molecular weight oligomers of adiponectin [100], a mechanism that reduces insulin sensitivity.

**Figure 4:**
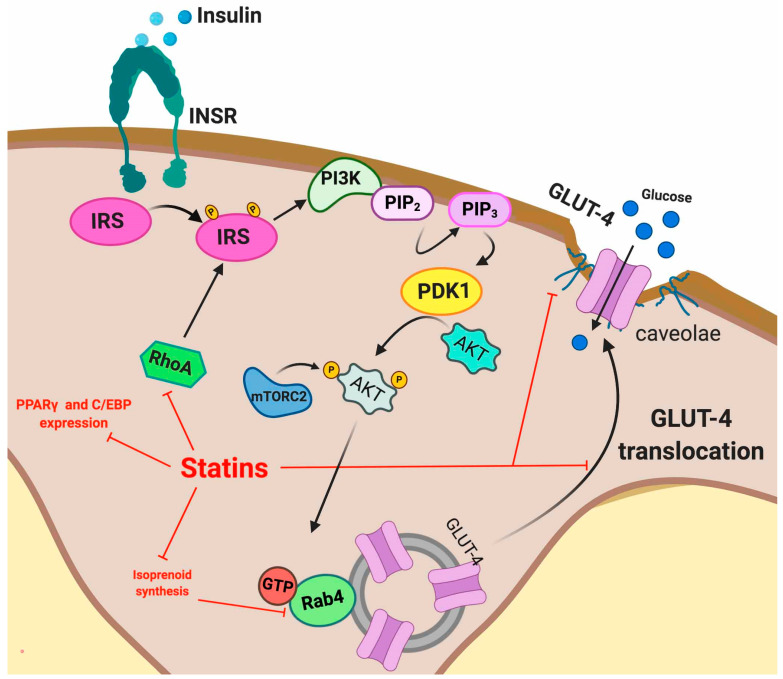
Intracellular actions of statins in adipocytes. Red lines indicate the mechanisms affected by statins.

Interestingly, statins also affect the preadipocyte to adipocyte differentiation process. The mechanism underlying this effect is likely the lack of secretion of insulin-sensitizing hormones. It has been shown that this is caused by a decrease in the expression of PPARγ (peroxisome proliferator–activated receptor γ) and C/EBP (CCAAT/enhancer-binding protein) transcription factors [56]. The adverse effects of statins are summarized in Figure 4.

#### 5.2.2. Skeletal Muscle

Skeletal muscle is the major tissue consuming most of the glucose that enters circulation [101], and any impairment in glucose uptake by this tissue may result in T2DM development. GLUT-4 mediates glucose transport into skeletal muscle cells, representing a key factor for blood sugar control [102]. As indicated above, insulin binding to INSR causes Akt activation [103] and translocation of GLUT-4 containing vesicles to the plasma membrane, thus facilitating the transport of glucose [104,105,106]. Although the mechanism of statin induced T2DM is not completely understood, there are both in vivo and in vitro studies that shed some light on this phenomenon in skeletal muscle. Some of the mechanisms that have been previously described are statin-mediated inhibition of insulin stimulated glucose uptake, impairment of intracellular signaling of the INSR and thereby of the Akt/mTOR pathway, or an excess of FFA accumulation in skeletal muscle as a consequence of HMG-CoA reductase inhibition.

In support of a role for a statin-induced insulin resistance in skeletal muscle, a decreased GLUT-4 expression has been found in L6 myotubes after simvastatin treatment [107]. Alternatively, it has been more recently shown that atorvastatin diminishes GLUT-4 translocation to the plasma membrane without affecting total GLUT-4 protein expression in C2C12 myotubes [108]. Assessment of the mechanism of simvastatin- or atorvastatin-associated impairment of glucose transport into myotubes suggests that impaired intracellular signaling of the INSR pathway also plays an important role. Indeed, Sanvee et al. [109] have shown that in C2C12 myotubes, simvastatin inhibits both INSR and mTORC2 function leading to impaired Akt activation and decreased translocation of GLUT-4 and consequently, reduced glucose uptake into skeletal muscle [109]. This deficient GLUT-4 translocation is likely caused by impaired Akt-mediated phosphorylation of GSK3β. Additionally, they show that simvastatin treatment induces higher plasma glucose levels in mice despite increased insulin plasma concentrations, consistent with insulin resistance [109]. The sequence of events leading to diminished glucose uptake induced by simvastatin starts with impaired phosphorylation of INSR, specifically the β-chain, which is considered to be essential for action of the receptor [101]. This results in deficient phosphorylation of Akt, which needs to be phosphorylated at both Thr308 (through the insulin signaling pathway) and Ser473 (by mTORC2) to become fully active [101]. Simvastatin treatment significantly impaired only the phosphorylation of Akt Ser473 due to an impaired phosphorylation of mTOR, one of the mTORC2 constituents [109,110]. Since Akt requires both phosphorylations to be fully active, it is then unable to activate glycogen synthase kinase 3β (GSK3β), which is involved in the translocation of GLUT-4 to the plasma membrane. Decreased GSK3β phosphorylation in the setting of simvastatin at least partially explains impaired translocation of GLUT-4 to the plasma membrane.

Another adverse effect related to statin-induced T2DM and, similar to in adipocytes, is deficient prenylation of RabGTPases, which has been suggested to lead to impaired GLUT-4 translocation [111]. Decreased intracellular cholesterol concentration is also considered a leading mechanism for impaired GLUT-4 translocation [108].

**Figure 5:**
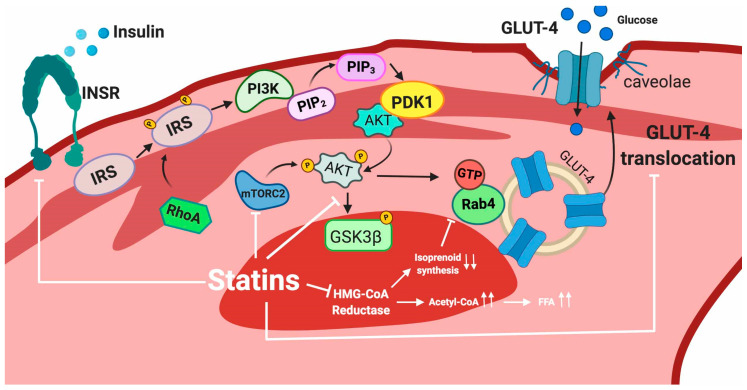
Intracellular actions of statins in muscle cells. White lines indicate the mechanisms affected by statins.

Alternatively, it has been suggested that simvastatin may cause insulin resistance through a novel fatty acid based mechanism independent of its cholesterol lowering effects. In their study, Kain et al. hypothesized that by blocking HMG CoA reductase, simvastatin may lead to accumulation of acetyl CoA, a precursor of fatty acid synthesis that can promote an intracellular build-up of fatty acids. The resulting excess accumulation of FFA in skeletal muscle may inhibit glucose uptake by reducing GLUT translocation [112,113]. The adverse effects of statins in muscle cells are summarized in Figure 5.

#### 5.2.3. Liver

The liver plays a central role in glucose homeostasis and is exquisitely sensitive to insulin. In fact, insulin regulates many hepatic metabolic pathways ranging from the glucose output to lipid synthesis. Therefore, impairment of hepatic insulin sensitivity is rapidly reflected in glucose homeostasis and triglyceride levels. Emerging evidence has demonstrated that statin treatment is associated with worsening glycemic control in the liver [114]. Several mechanisms possibly involved with the effect of statins on glucose metabolism in the liver are summarized below.

Statin therapy is associated with a small increment in fasting blood glucose levels [115]. It has been shown that statins can stimulate endogenous glucose production by activation of phosphoenolpyruvate carboxykinase (PEPCK) and glucose-6-phosphatase (G6Pase) [116,117], the major rate-limiting gluconeogenic enzymes in human liver cells. The elevation of hepatic gluconeogenesis contributes to hyperglycemia, which is characteristic of insulin resistance and T2DM.

**Figure 6:**
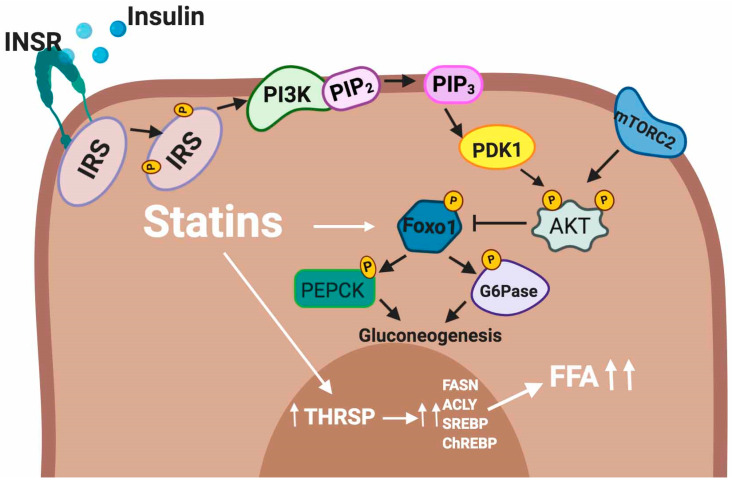
Intracellular actions of statins in hepatocytes. White lines indicate the mechanisms affected by statins.

Regarding FFAs, it has been shown that an excess of FFA accumulation in liver cells can contribute to the development of T2DM [118,119]. Interestingly, atorvastatin and rosuvastatin treatment upregulates thyroid hormone-responsive spot 14 protein (THRSP) expression, which is a small protein predominantly expressed in lipid-producing tissues such as those found in the liver. THRSP has been implicated as a regulator of the lipogenic processes by controlling the expression of lipogenic genes such as fatty-acid synthase (FASN), ATP citrate lyase (ACLY) SREBP and ChREBP [120,121] or their activity [122]. The adverse effects of statins in the hepatocytes are summarized in Figure 6.

### 5.3. MicroRNAs and Impact of Statin Therapy on microRNA Expression Profile

MicroRNAs (miRs) are small (22 nucleotide) noncoding regulatory RNAs, which act as post-transcriptional regulators of gene expression [123,124]. miRs usually silence gene expression through mRNA degradation or sequestration of the target mRNA from translation machinery [125]. It has been shown that miRs are involved in many biological processes including insulin expression, skeletal muscle adaptation to elevated glucose, insulin sensitivity and glucose stimulated insulin secretion (GSIS) [126]. It has been shown that miRs likely mediate the pleiotropic effects of statins via modulation of lipid metabolism, enhancement of endothelial function, inhibition of inflammation, improvement of plaque stability and immune regulation. More specifically, miRs appear to regulate the fine-tuning of cellular phenotypes rather than serving as molecular on–off switches [127].

Statin therapy has been found to affect the expression of several miRs, which play a central role in the regulation of lipid and glucose metabolism [128] and that are associated with development of T2DM.

#### 5.3.1. miR Modulation of Cholesterol and Lipid Homeostasis

miR-33a and miR-33b are encoded within the introns of the Srebp2 and Srebp1 genes, respectively, and modulate intracellular cholesterol and fatty acid homeostasis together with SREBP2 and SREBP1 [129,130,131,132]. Specifically, miR-33a targets genes involved in cholesterol export, inhibits ABCA1 and ABCG expression [130,131,132] and participates in the regulation of HDL levels in vivo. On the other hand, miR-33b modulates metabolic pathways related to of fatty acid metabolism [129,133]. Importantly, both miR-33a and miR-33b participate in the regulation of fatty acid metabolism and are involved in the regulation of lipid and glucose metabolism [129]. miR-33 also negatively affects IRS2 expression thereby affecting insulin signaling [129]. Collectively, both isoforms of miR-33 participate in the regulation of relevant pathways that impact the primary risk factors of insulin resistance.

It has been demonstrated that simvastatin and atorvastatin induce expression of miR-33a in the liver [134] thus suggesting a link between reduced insulin secretion and, ultimately, the development of statin-induced T2DM. miR-33a is an important regulator of ABCA1 and their expression levels are inversely proportional in β-cells [132,135]. miR-33a-mediated downregulation of ABCA1 can also alter islet cholesterol homeostasis and impair insulin secretion thus leading to β-cell dysfunction [136,137]. However, additional studies are needed to further confirm the presence of a causal relationship between statin treatment and miRs in T2DM. Several studies have shown that statin treatment can upregulate miR-33b expression thus suggesting that statins could interfere fatty acid metabolism [138,139].

Recently, the miR-27 family (miR-27a and miR-27b) has emerged as a new key regulator of cholesterol and lipid homeostasis [140,141,142]. Interestingly, the miR-27 family has been shown to be upregulated in a dose-dependent manner by simvastatin in HepG2 cells. Alvarez et al. demonstrated that miR-27a directly decreases both LDLR RNA and protein levels by binding to the 3′UTR of the *LDLR* mRNA [143]. Moreover, miR-27a also decreases LDLR expression indirectly through upregulation of PCSK9. They suggest that the potential binding site for miR-27a at position -1671 bp relative to the transcription start site of *PCSK9* may be responsible for the upregulation of PCSK9. In addition to the direct and indirect downregulation of LDLR levels, miR-27a also indirectly affects LDLR efficiency through a mechanism in which miR-27a targets the 3′UTR sequence of two genes in the LDLR pathway: LRP6 and LDLRAP1 by downregulating their expression [143]. Both proteins are necessary for correct binding to clathrin and thus are essential for efficient endocytosis of the LDLR-LDL-C complex [144,145,146]. Therefore, in addition to decreasing LDLR levels at the plasma membrane, miR-27a may also negatively affect LDLR efficiency. Deregulation of miR-27a has been reported in T2DM [147]. Specifically, it has been shown to be upregulated in adipose tissue and in 3T3-L1 adipocytes exposed to increased glucose concentration [147].

#### 5.3.2. Modulation of Hepatic Glucose Production

A vast number of miRs have been described to modulate glucose homeostasis through various mechanisms, leading to the question of whether some of them may potentially be involved in statins’ diabetogenic effects. Specifically, it has been demonstrated that a direct effect of statins on hepatic glucose production is mediated by upregulation of the miR-183/96/182 cluster by modulating the expression of gluconeogenic enzymes [148]. It has been shown that incubation of hepatocytes with atorvastatin, simvastatin or pravastatin upregulates the expression the key gluconeogenic enzymes PEPCK and G6Pase [117,149]. The statin-mediated effects involve miR-183/96/182-mediated downregulation of the transcription factor 7-like 2 (TCF7L2), which modulates hepatic and peripheral glucose metabolism and whole body glycemic control [148]. In regards to gluconeogenesis, TCF7L2 also reduces hepatic gluconeogenesis likely by decreasing the transcriptional activity of positive regulators of PEPCK and G6PC [150,151,152,153,154]. These results suggest that patients under long-term statin treatment would have persistently elevated expression of the miR cluster and lead to sustained activation of the gluconeogenic pathway, ultimately contributing to T2DM.

#### 5.3.3. Modulation of the Insulin Signaling Pathway

As mentioned above, the activation of INSR by insulin leads to structural rearrangements in the receptor leading to autophosphorylation at tyrosine residues. Within the cell, phosphorylation levels are tightly regulated by protein phosphatases, in this case protein tyrosine phosphatases (PTPAses). These PTPAses negatively modulate insulin signaling by removing phosphate groups from tyrosine residues of the cytoplasmic domain of INSR. Specifically, protein tyrosine phosphatase non-receptor type 1 (PTPN1) has been predicted as an miR-146a target and the expression of PTPN1 is inversely correlated with miR-146a both in the skeletal muscle and in the liver of a T2DM rat model [155]. Of note, the role of miR-146a has been widely investigated in human T2DM pathogenesis and several studies report that it is downregulated in whole blood, plasma and some peripheral tissues [156]. Notably, it has been shown that simvastatin treatment also downregulates mir-146a expression after 6 months of therapy [157].

As mentioned above, IRSs link INSR activation to insulin metabolic effects through the intermediate modulation of the PI3K/PDK1/Akt pathway. It has been described that expression levels of IRS1 are modulated by miR-145 in hepatocytes [158] whereas in mice, upregulation of miR-145 in the liver leads to insulin resistance [159]. Atorvastatin treatment has also been shown to differentially upregulate miR-145 and modulates PI3K/Akt signaling pathway [160].

In hepatocytes, miR-33a and miR-33b have been reported to modulate fatty acid and cholesterol metabolism as well as insulin signaling by targeting IRS2 [129,161]. In one study, miR-33b overexpression in the Huh7 human hepatocytes cell line resulted in reduced Akt and ERK phosphorylation secondary to IRS2 down-regulation [129].

Additional Akt-downstream kinases and phosphatases represent major regulators of insulin signaling. Direct inactivation of AKT is mediated by protein phosphatase 2a (PP2A) [162]. PP2A activity has been shown to be increased in primary rat hepatocytes in insulin resistance condition [163]. Interestingly, insulin resistant Zucker Diabetic Fatty rats, PP2A mRNA is increased in liver, muscle and adipose tissue, thus suggesting a role for the phosphatase in deregulating insulin signaling in T2DM [163]. Importantly, PP2A expression is modulated by several miRs, among them miR-155 [164], whose expression has been found to be altered in T2DM. In addition, high dose rosuvastatin treatment has been shown to reduce the relative levels of serum miR-155 and therefore could lead to increased expression and activity of PP2A [165].

## 6. Differences in Diabetogenic Effects between Hydrophilic and Lipophilic Statins

As indicated in previous sections, lipophilic statins (atorvastatin, simvastatin, lovastatin, fluvastatin and pitavastatin) may be more diabetogenic than hydrophilic statins (pravastatin and rosuvastatin) as they can more readily penetrate extrahepatic cell membranes such as β-cells, adipocytes and skeletal muscle cells. Conversely, hydrophilic statins (e.g., pravastatin) are more hepatocyte specific and less likely to enter β-cells or adipocytes [29]. Indeed, a high hepato-selectivity translates into minimal interference with cholesterol metabolism in tissues other than the liver and consequently to a lesser diabetogenicity [29,30,56]. Several studies have shown that the detrimental effects of statins are dose and potency dependent and primarily related to their lipophilicity [5,14,41,47,166].

While lipophilic statins have negative effects on pancreatic β-cell function, for hydrophilic statins such as pravastatin, neutral or improving effects have been observed [40,41,167]. As mentioned in Section 4, it has been reported that statins can inhibit glucose-induced cytosolic Ca^2+^ signaling and insulin secretion by blocking L-type Ca^2+^ channels in β-cells. These inhibitory potencies may be particularly evident for the lipophilic rather than the hydrophilic statins [41,166,168]. Indeed, unlike hydrophilic statins, the lipophilic ones have a strong affinity for the cell membrane, and therefore have easier access to the intracellular space [168]. In this context, statins may inhibit the endogenous metabolic pathways described in Section 5.1 that are associated with glucose-stimulated insulin secretion, including endogenous cholesterol synthesis [73,166] and Ca^2+^-dependent insulin responses to glucose [168]. It has been shown that atorvastatin (lipophilic) but not pravastatin (hydrophilic) affects insulin release and mitochondrial metabolism due to the suppression of antioxidant defense system and induction of ROS production in pancreatic β-cell models [169].

As described in Section 5.2.1, Section 5.2.2 and Section 5.2.3, GLUT-4 mediates insulin-stimulated glucose uptake [86] in a process that requires fusion of the transporter with the plasma membranes facilitated by IRS-1 and several kinases [86,170,171]. The small GTP-binding proteins are also key players in this process [22,86] and they require isoprenylation by mevalonate products for their association with the cell membranes. The statin-mediated inhibition of the synthesis of the above products increases insulin resistance in parallel with the mevalonate synthesis inhibitory capacity [21,172]. Furthermore, several other processes involved in the GLUT-4 signaling pathway may be inhibited by statins. These include IRS-1, insulin receptor β subunit, and Akt phosphorylation [22,166]. It has been suggested that these effects are relevant only for lipophilic statins (e.g., atorvastatin and simvastatin), but not for hydrophilic statins (e.g., pravastatin) [22,166]. The capacity of the former to enter adipocytes through passive diffusion can help explain this difference.

## 7. Conclusions

Taken together, the studies described in this review, ranging from clinical studies to in vivo and in vitro experimental results, confirm and reinforce the diabetogenic effect of statins. Although a number of questions remain unanswered, the available evidence supports that statins do increase the chances of T2DM with some statins being more strongly related (e.g., simvastatin, rosuvastatin and atorvastatin) than others (e.g., pravastatin). Intense research is currently going on to elucidate the mechanisms of statin induced T2DM at the molecular level. In light of the evidence from multiple observational studies, it is important to emphasize that there is still a favorable risk–benefit ratio for statin therapy, due to the large reduction in cardiovascular risk, despite the adverse effect of T2DM development. Overall, the risk of incident diabetes mellitus with statin therapy is present but largely outweighed by the actual cardiovascular benefits [16]. Thus, statins should be continued in patients in whom these drugs are prescribed due to high or very high CVD risk, despite the risk of T2DM development until they achieve the target LDL-C levels. Before initiation of statin therapy the risk of diabetes should be assessed [8,16,173]. Statin-treated patients at high risk of developing diabetes should be monitored for changes in blood glucose and HbA1c levels, and preventive lifestyle modification should be introduced. If diabetes develops, it should be managed according to the guidelines [16]. Patients should be educated regarding the risk of incident diabetes mellitus with statins as with other risk–benefit of all therapies [174]. Lifestyle modification should be encouraged to lower cardiovascular risk and that for developing T2DN [175] and national guidelines should be used to manage diabetes mellitus [176,177].

Several mechanisms through which statin treatment causes β-cell dysfunction and insulin resistance in peripheral tissues have been identified. Specifically, these the diabetogenic effects are related both to the dose and statin class. In addition, miRs are glucose homeostasis regulators through the specific modulation of insulin signaling components. Growing evidence indicates that statin modulation of miRs expression may also be another mechanism through which statins increase the risk of T2DM. A multifactorial combination of these effects is what most likely contributes to the diabetogenic effects of statins described here. Clinically, these findings should encourage clinicians to consider diabetes monitoring in patients receiving statin therapy in order to ensure early diagnosis and appropriate management. Ultimately, since the risk of statin-induced T2DM is still being characterized, and the efficacy of statins in preventing CVD is very well documented, statins remain a first line treatment for prevention of CVD.

## Abbreviations

Cav-1	Caveolin 1
CVD	Cardiovascular disease
FASN	Fatty-acid synthase
FFA	Free fatty acids
G-proteins	Small GTP-binding proteins
G6Pase	Glucose-6-phosphatase
GLUT-4	Insulin-responsive glucose transporter 4
GSIS	Stimulated insulin secretion
HMG-CoA	3-hydroxy-3-methyl-glutaryl coenzyme-A
INSR	Insulin receptor
IRS	Insulin receptor substrates
LDL-C	LDL cholesterol
LDLR	LDL receptor
miR	MicroRNA
PEPCK	Phosphoenolpyruvate carboxykinase
PP2A	Protein phosphatase 2a
PP2CA	Protein phosphatase 2CA
PTPAses	Protein tyrosine phosphatases
PXR	Pregnane X receptor
RCT	Randomized control trials
SGK2	Serum/glucocorticoid regulated kinase 2
T2DM	Type 2 diabetes mellitus
